# A Very Low CP Level Reduced Diarrhoea and Productivity in Weaner Pigs, but No Differences between Post-Weaning Diets Including Soybean Meal or Soy Protein Concentrate Were Found

**DOI:** 10.3390/ani11030678

**Published:** 2021-03-04

**Authors:** Julie C. Lynegaard, Niels J. Kjeldsen, Julie K. Bache, Nicolai R. Weber, Christian F. Hansen, Jens Peter Nielsen, Charlotte Amdi

**Affiliations:** 1 Department of Veterinary and Animal Sciences, University of Copenhagen, DK-1870 Frederiksberg C, Denmark; julie.lynegaard@sund.ku.dk (J.C.L.); jpni@sund.ku.dk (J.P.N.); 2 Pig Research Centre, Danish Agriculture and Food Council, SEGES, DK-1609 Copenhagen V, Denmark; njk@seges.dk (N.J.K.); juk@seges.dk (J.K.B.); nirw@seges.dk (N.R.W.); cfha@seges.dk (C.F.H.)

**Keywords:** amino acids, antibiotics, growth performance, post-weaning diarrhoea, protein, swine

## Abstract

**Simple Summary:**

Diarrhoea is a challenge after weaning in pigs, and medicinal zinc oxide has been used to decrease this problem. Additionally, soy protein concentrate improves protein digestion and thereby increases growth compared to soybean meal. The aim was to test the effect of different low-protein diets on diarrhoea and productivity. This study demonstrates that a diet with very low-protein levels supplemented with amino acids decreases diarrhoea similar to zinc oxide, but also limits growth performance. Moreover, the study found no effect of low-protein diets with different protein sources. Based on these results, there is potential in feeding extremely low-protein diets to weaned pigs as a tool to decrease diarrhoea and thereby antibiotics usage. Additionally, this study presents an opportunity to use both soy protein concentrate and soybean meal as the main protein source, without affecting pig health and productivity.

**Abstract:**

Soy protein concentrate improves nutrient utilization and growth performance compared to soybean meal, and diets with a low crude protein (CP) level decreases diarrhoea. The objectives were to (1) test a low CP diet based on different soy products, and (2) to test a very-low CP diet (15.1%) with amino acids (AA) on diarrhoea and productivity. A total of 5,635 weaned pigs (~28 days), were assigned to five dietary treatments; PC (positive control): Standard CP levels (192, 189, 191 g/kg CP) with 2500 ppm ZnO; NC (negative control): Same as PC without ZnO; SP (Soy protein concentrate): Low CP levels (176, 174, 191 g/kg CP); SB (Soybean meal): Low CP levels (177, 176, 191 g/kg CP); and XLA (X-low CP + AA): Very low CP levels (154, 151, 191 g/kg CP) with AA. The PC and XLA diets reduced diarrhoea by 41 and 61%, respectively, compared to the NC group, while no difference between SB and SP were observed. The XLA diet reduced feed intake and daily gain compared with PC and NC, where SP, SB, and XLA had a poorer feed conversion compared with PC. Conclusively, the SP and SB low-protein diets did not reduce diarrhoea or growth performance, whereas the XLA diet decreased both diarrhoea and performance.

## 1. Introduction

Medicinal zinc oxide (ZnO) is well-documented to have a reducing effect on diarrhoea in weaned pigs two weeks post-weaning [1,2]. However, as medicinal ZnO has been banned in the EU from June 2022, an increased diarrhoea frequency resulting in higher antibiotics (AB) use is feared. A low crude protein (CP) allocation is also well-documented to reduce post-weaning diarrhoea [3,4,5], as a high-protein diet increases the amount of undigested protein available for fermentation in the colon, which may lead to proliferation of pathogenic bacteria in the gastrointestinal tract [5,6]. The inclusion of free amino acids (AA) in low CP diets is essential, as the low CP allocation results in an undersupply of essential AA and reduces productivity [4,7].

Recently, a study demonstrated that a low CP diet reduces diarrhoea by 30% in the nursery period compared with pigs fed standard CP levels without medicinal ZnO [4]. At the same time, growth performance decreased with the low CP levels even when supplemented with essential AA (Lys, Met, Thr, Trp, and Val). It was therefore expected that the remaining essential AAs became limiting for growth. The CP reduction in Lynegaard et al. [4] was obtained by lowering the costly protein sources—fishmeal, potato protein concentrate and soy protein concentrate (SPC)—thereby maintaining the level of soybean meal (SBM).

Soybean products are an excellent source of protein for pigs as the AA profile complements that of cereal grains [8]. However, SBM contains several potential antinutritional factors, including trypsin inhibitors, lectins, saponins, and antigenic proteins that may reduce digestibility and growth performance in young pigs as well as increase post-weaning diarrhoea [8,9,10]. SPC, on the other hand, has fewer antinutritional factors and a lower non-starch polysaccharides content than SBM and therefore contains protein which is more digestible for the weaned pig [8,11]. Consequently, substitution of SBM with SPC could be expected to result in improved nutrient utilization and growth performance [8,12,13]. On the other hand, a recent Danish trial demonstrated no differences in diarrhoea frequency between pigs fed SPC or SBM [14]. However, there is still a general concern among Danish pig producers that SBM reduces the average daily feed intake (ADFI) and increases diarrhoea frequency post-weaning compared to SPC, and consequently is not the most optimal protein source for newly weaning pigs. Therefore, the current study was carried out with low CP levels of different protein sources (SBM and SPC) supplemented with essential AA (Lys, Met, Thr, Trp, Val), and one dietary group included additional essential AA (Leu, Ile, Phe, His and Tyr, Evonik, Essen, Germany).

The primary aim of this study was to test the effect of a low dietary CP level obtained by different protein sources (SPC vs. SBM) supplemented with the new Danish recommendations for AA without medicinal ZnO on diarrhoea frequency and growth performance. The secondary aim was to test whether a very low dietary CP level supplemented with additional essential AA without medicinal ZnO could reduce diarrhoea similar to a standard post-weaning diet with medicinal ZnO, without limiting performance.

## 2. Materials and Methods

The study was carried out at the Danish Pig Research Centres experimental station in Denmark and complied with the laws and regulations for the humane care and use of animals in research [15].

### 2.1. Experimental Design, Animals and Housing

The study was designed as a complete randomized block feeding trial to test different protein levels and sources for weaned pigs, where a block was comprised of six pens with different dietary treatment groups. The study included a total of 5,635 weaned pigs (Duroc x (Danish Landrace × Yorkshire), DanBred, Denmark) from two commercial Danish piggeries. Newly weaned pigs entered the trial on a weekly basis and were randomly allocated in a complete block design to one of five dietary treatments balanced by weight (small, medium, large) and sex. The trial included 80 blocks (replicates) with six pens in each (the NC group had twice as many pens), divided into 22 weekly batches (every batch were divided onto two or three blocks). The pigs entered the trial unit at ~28 days of age with a bodyweight (BW) between 5.5 kg and 9 kg. Pigs were divided in pens so that the pens within a block (six pens) had a maximum BW difference of ±0.25 kg per pig. The pigs were included in the trial until they reached a BW of approximately 30 kg or a maximum of seven weeks from entry.

The experimental station was divided into eight sections. Four of these had 18 pens of 4 m^2^ with up to 10 pigs and four sections had 12 pens of 5 m^2^ with up to 15 pigs. Each pen had 2 m^2^ of solid floor and the remaining floor was slatted. The pens had a cover over about 40% of the floor area and each pen had a water dispenser and an individual Spotmix feeder (Schauer Agrotronic GmbH, Prambachkirchen, Austria). The pigs were vaccinated against Porcine Circovirus type 2 with 0.5 mL Circovac (Ceva Animal Health A/S) at arrival.

### 2.2. Dietary Treatments

The five dietary treatments included two control groups and three protein strategies with different protein sources divided into a three-phase feeding plan, supplemented with essential AA (Lys, Met, Thr, Trp, Val) (Table 1). The treatments were: standard CP levels (192, 189, 191 g/kg CP) with the AA-recommended profile from 2018 [16] and allocated 2500 ppm of medicinal zinc oxide in phase 1 (PC = positive control); similar to PC but without medicinal ZnO (NC = negative control); low CP levels (176, 174, 191 g/kg CP), with SPC as the main protein source (SP = soy protein concentrate/Vilosoy) and with the AA recommended profile from 2019 [17]; low CP levels (177, 176, 191 g/kg CP) with SBM as the main protein ingredient (SB = soybean meal) and with the AA recommended profile from 2019 [17]; and lastly, very low CP levels (154, 151, 191 g/kg CP), with the AA recommended profile from 2019 [17] and supplemented with additional AA in phase 1 and 2 (XLA = x-low-protein + Leu, Ile, Phe, His and Tyr, Evonik, Essen, Germany).

The main ingredients were wheat and barley, and the diets were calculated to be isoenergetic. Dietary ingredients and diet composition are summarized in Supplementary Tables S1–S3. The analysed AA profiles can be viewed in Table 1 and the expected amounts are summarized in Supplementary Table S4. The PC and NC diets were formulated with increasing SBM in the three phases and decreasing SPC (7, 14, 21% and 6.5, 2.9, 2.1%, respectively). The SP diet was formulated with an increasing SBM inclusion and a decreasing SPC inclusion (0.5, 6, 22.5% and 7.5, 2.6, 0.5%, respectively), whereas the SB diet had an increasing SBM inclusion and a decreasing SPC (7.0, 14.0, 22.5% and 2.2, 0.85, 0.5%, respectively). Lastly, the XLA diet had an increasing level of SBM (0.5, 2.2, and 22.5%) and only included 0.5% SPC in phase 3.

### 2.3. Feeding and Feeding System

Pigs received their dietary treatments from the day of entry (day 1) until seven weeks post-weaning at the end of the trial (~30 kg) and the diets were based on wheat, barley and SBM or SPC (Vilosoy, Vilomix, Sjølund, Denmark). The diets were formulated as dry pelleted feed and pigs had ad libitum access to feed. The dietary treatments were allocated in a three-phase feeding plan based on both days and BW: phase 1 (days 1 to 11), phase 2 (day 11 to ~BW of 15 kg) and phase 3 (~BW of 15 to ~30 kg or end of the trial). The feed was changed gradually over a three-day period, starting at the end of each phase. The shift between phase 1 and 2 was set at day 11 post-weaning to ensure no medicinal ZnO in the diet after day 14 post-weaning. The pen weight was tested regularly to make the feed change from phase 2 to 3 at an average pen BW of 15 kg (±1.5 kg).

### 2.4. Feed Manufacturing and Analysis

The five dietary treatments were manufactured by Danish Agro (Sjølund, Denmark) four times throughout the experimental period. Three feed samples were extracted from each diet during manufacturing based on the TOS principles [18]. The feed samples were analysed at a commercial feed testing laboratory (Eurofins Steins Laboratory A/S, Vejen, Denmark).

### 2.5. Recordings and Sampling

Diarrhoea treatments were the primary parameter and were recorded on both an individual and pen basis. Diarrhoea treatments were performed by the staff according to the veterinarian’s instructions, by recognizing the signs of diarrhoea disease—perineal faecal staining, sunken eyes, hollow lumbar region and lethargy. The first two pigs in a pen diagnosed with clinical diarrhoea were individually treated with AB for three days with Linco-Spectin^®^ or Clamoxyl^®^ (Orion Pharma Animal Health, Copenhagen, Denmark) or Streptocillin^®^ Vet (Boehringer Ingelheim Animal Health, Copenhagen, Denmark). When more than two pigs within a pen required AB treatments for diarrhoea, the entire pen was orally treated with a mix of AB and wheat bran, which was mixed directly into the feed for five days (Clamoxyl^®^ Vet, Orion Pharma Animal Health, Copenhagen, Denmark from day 1 to 14 and thereafter Doxylin^®^ Vet., Salfarm Denmark A/S, Kolding, Denmark).

Performance parameters were recorded at a pen level as ADFI, average daily gain (ADG) and feed conversion ratio (FCR). The BW of all pigs within a pen were recorded on a scale (Bjerringbro vægte APS, Bjerringbro, Denmark) at entry to the experimental station (day 1), at day 11, and at ~15 and ~30 kg. Pens were therefore weighed continuously to check when the average BW was 15 and 30 kg, respectively. The BW and day were recorded when the desired BW was reached. Feed consumption per pen was recorded daily for each dietary treatment. If pigs were deemed unfit to continue the trial, they were removed from the pen and BW was recorded.

Faecal samples were collected from pens immediately before a diarrhoea pen treatment. The faecal samples were collected by swiping a gloved hand over the slatted floors and pooled into a sealed plastic container. Samples were stored in a −20 °C freezer until analysis for the detection of pathogens (L. *intracellularis*, B. *pilosicoli*, E.coli F4 and F18) at Kjellerup Laboratory [19]. If a faecal sample had a bacterial count above 35,000 per gram of faeces, the result was interpreted as bacterial pathogenic diarrhoea. [20].

### 2.6. Calculations and Statistical Analysis

All statistical analyses were conducted using SAS Enterprise Guide 7.1 (SAS Inst. Inc., Cary, NC, USA) with the pen as the experimental unit. Statistical significance was accepted at *p* < 0.05 and a *p*-value between 0.05 and 0.10 was considered a tendency.

Diarrhoea pen treatments and faecal pen samples were analysed using a logistic regression model, with the dietary treatment as a fixed effect, BW at entry as a covariate, block as a random effect and pen as the experimental unit. The diarrhoea treatment days per pig was analysed using a logistic regression model, with the dietary treatment as a fixed effect, BW at entry as a covariate, block as a random effect, and pig as the experimental unit. In these models, the effect of dietary treatment was tested against the NC group and no further pairwise comparisons were performed.

Performance parameters (ADG, ADFI and FCR) were analysed using a linear mixed model, with a fixed effect of dietary treatment, BW at entry as a covariate, block as random effects and pen as the experimental unit. In these models, all dietary treatments were compared and 15 pairwise comparisons were performed with a Tukey correction.

Additionally, analyses were performed with BW at entry (small, medium, and large) as a fixed effect. Diarrhoea treatments were analysed using a logistic regression model, with entry BW and dietary treatment as fixed effects and block as random effects. Performance parameters were analysed using a linear mixed model, with BW at entry and dietary treatment as fixed effects, and block as random effects.

## 3. Results

### 3.1. Feed Analysis

The analysed CP and AA content of the diets are shown in Table 1 and the actual dietary concentrations of CP and AA in relation to the expected values are summarized in Table 2. In general, there was an undersupply of Met, His, Ile, Leu, and Met + Cys, and Val was oversupplied for all dietary treatments in phase 1 and 3, whereas Met, Ile, Leu, and Met + Cys was undersupplied for all treatments in phase 2. Deviations of up to 5% are accepted, but His, Met, and Met + Cys were severely deficient.

### 3.2. Diarrhoea Treatments

**Figure 1:**
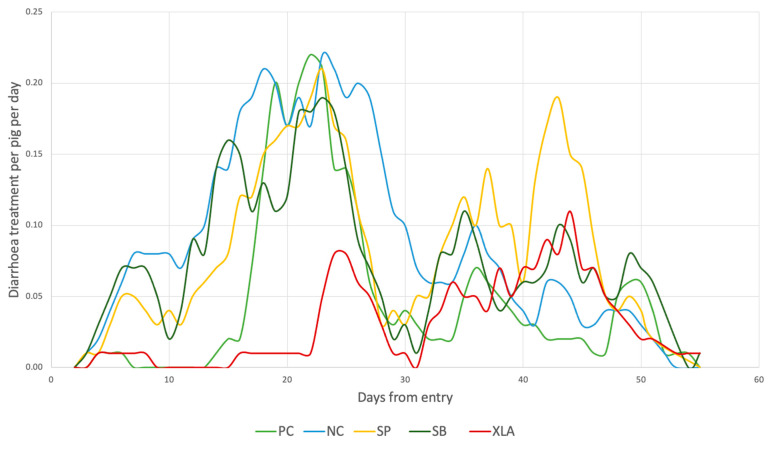
The number of diarrhoea treatments per pig per day; PC = positive control group with medicinal zinc oxide; NC = negative control group without medicinal zinc oxide; SP = soy protein concentrate; SB = soybean meal; XLA = X-low-protein + amino acids.

**Figure 2:**
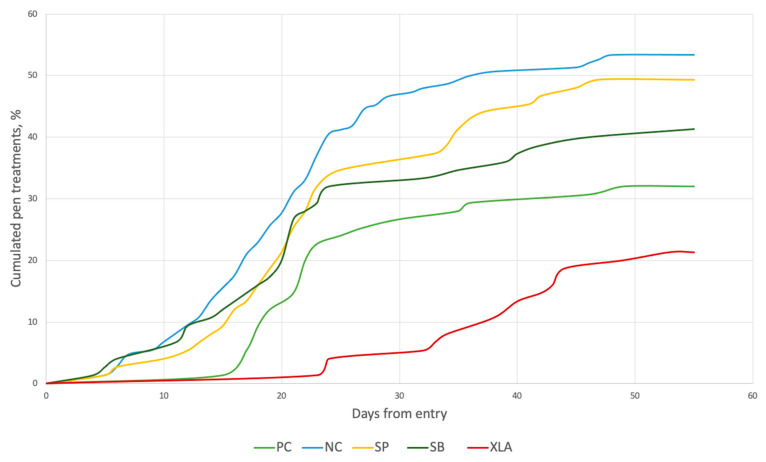
Cumulated pen diarrhoea treatments in weaned pigs; PC = positive control group with medicinal zinc oxide; NC = negative control group without medicinal zinc oxide; SP = soy protein concentrate; SB = soybean meal; XLA = X-low-protein + amino acids.

Diarrhoea treatments per pig can be seen in Figure 1 and accumulated diarrhoea pen treatments in Figure 2. There was a peak in diarrhoea treatments per pig starting around day 10 until day 30 in the NC, SP, and SB pigs, whereas the PC and XLA pigs only had a small peak around day 24. Additionally, all dietary treatments had a diarrhoea peak late in the trial, at around day 35. In groups NC, SB, and SP, the pen treatments started at day 5 while the PC pigs did not receive any diarrhoea pen treatments in the 14 days where the medicinal ZnO was included in the feed. For the XLA pigs, almost no diarrhoea pen treatments were given before day 24.

The effect of dietary group on diarrhoea treatments during the trial period are summarized in Table 3. In the overall trial period, the PC pigs had ~40% fewer diarrhoea pen treatments and the XLA pigs had ~61% fewer diarrhoea pen treatments compared with the NC pigs (Diarrhoea treated pens; PC = 31.8%, XLA = 21.1% compared to NC = 53.5%, *p* < 0.05). This difference was mostly caused by a difference in diarrhoea pen treatments during phase 2 where the PC had 40% fewer diarrhoea pen treatments and XLA pigs had ~91% fewer diarrhoea pen treatments compared with the NC pigs (Diarrhoea treated pens; PC = 26.7%, XLA = 4.0% compared to NC = 44.6%, *p* < 0.05). During phase 2, both the PC and XLA pigs but also the SP and SM pigs had significantly fewer treatment days per pig compared with the NC pigs (Diarrhoea treatment days per pig; PC = 1.82, SP = 2.15, SB = 1.98, XLA = 0.38 compared to NC = 3.05, *p* < 0.05).

### 3.3. Results from Pen Faecal Samples

A total of 143 pen faecal samples were analysed for bacterial intestinal pathogens in the five dietary groups. The analysis revealed that 12 pens were positive for *L. intracellularis*, one pen was positive for *B. pilosicoli*, 27 pens had samples positive with E. Coli F4 and 26 pens were positive for E. Coli F18. No differences in detected pathogens were revealed between dietary groups (*p* > 0.05). In total, 35% of pen floor samples were positive for pathogens in the PC group, 46% in the NC group, 34% in the SP group, 45% in the SB group, and 33% of pen samples were positive for pathogens in the XLA group, but no differences were detected across dietary treatments (*p* > 0.05). In total, 59% of all pen faecal samples were negative for all four analysed pathogens.

### 3.4. Average Daily Gain, Feed Intake and Feed Conversion Ratio

Effect of dietary treatment on performance parameters during the nursery period are summarized in Table 4. The two low-protein dietary treatments (SP and SB) both resulted in a poorer FCR (1.44 and 1.45 kg/kg growth, respectively) compared to the PC pigs (1.41 kg/kg growth, *p* < 0.05) during the overall trial period. Whereas there was no difference (*p* > 0.05) in ADFI, ADG, and FCR between SP and SB during any of the three feeding phases. The XLA pigs had a lower ADFI of ~30 g/d and a reduced ADG of ~42 g/d during the overall trial period (*p* < 0.05) compared to PC and NC pigs, resulting in an impaired FCR (*p* < 0.05). The NC group had about 2% more pigs removed from the trial (*p* < 0.05) compared with the SP, SB, and XLA groups (Table 4).

### 3.5. Effect of Entry Bodyweight

At the beginning of the trial, pigs were divided between pens according to size (small, medium, and large), and the effect of entry BW on diarrhoea treatments and growth performance are displayed in Table 5. Pens with small pigs had ~26.8% fewer diarrhoea-treated pens compared to pens with large pigs (diarrhoea-treated pens; small = 32.7%, large = 45.4%, *p* < 0.001). A lower entry weight resulted in a reduced ADG from weaning to 30 kg (*p* <0.001) with an ADG of 451 g/day, 463 g/day, and 491 g/day for small, medium, and large pigs, respectively. Additionally, a lower entry weight was followed by a lower ADFI during the trial period (*p* < 0.001). On the other hand, pigs with a low entry weight had an improved FCR of 1.43 compared with an FCR of 1.45 in medium and large entry pigs (*p* = 0.008).

## 4. Discussion

By allocating pigs a low CP diet post-weaning, the amount of undigested proteins reaching the hindgut of the pigs and used for microbial fermentation of potentially toxic compounds resulting in diarrhoea, is limited [21,22]. At the same time, both SBM and SPC are exceptional sources of protein for pigs due to their high digestibility as well as their AA composition [8]. Full soybeans contain several ANFs, whereas the processing of SBM inactivates the heat labile ANFs, whereas SPC is processed to inactivate both heat labile and heat stabile ANFs [23]. For young pigs, SPC is therefore a common choice to increase digestibility and reduce the amount of undigested protein resulting in diarrhoea. However, the current study revealed no difference in diarrhoea pen treatments between pigs fed different protein sources (SBM and SPC) during the overall trial period when feeding a three-phase diet with a low CP level post-weaning (17.6%, 17.4% and 19.1%, in phase 1, 2 and 3, respectively). This is in agreement with a previous Danish study reporting no effect of protein source (SBM vs. Vilosoy, HP 300, AlphaSoy) on post-weaning diarrhoea when feeding similar CP levels in a three-phase diet (~18%, 18.5%, 19% CP, in the three phases) [14]. On the contrary, others have found a reducing effect of SPC on post-weaning diarrhoea compared with SBM in the immediate post-weaning period (21 to 49 days of age) [13]. However, in the study by Guzmán et al. [13], the protein levels were higher post-weaning (SPC = 20.9% CP and 22.3% CP vs. SBM = 21.8% CP, 21.0% CP and 21.8% CP) compared with the present study. As it is well-documented that a low CP level post-weaning reduces diarrhoea [7,24,25], it can be speculated that the positive effect of SPC on post-weaning diarrhoea, as seen in Guzmán et al. [13], only occurs at higher CP levels. Additionally, the inclusion of SBM was higher in their study (12%) post-weaning, compared with the current study, which only allocated 7% SBM in phase 1. The results therefore suggest that the presence of ANFs in SBM only reduces nutrient utilization and thereby affects diarrhoea post-weaning at higher CP levels than in the current trial.

Previous results by Heo et al. [2] found that medicinal ZnO (2500 ppm) reduces the incidence of diarrhoea 14 days post-weaning regardless of protein levels (251 g/kg vs. 192 g/kg CP). They further demonstrated that a low-protein diet (192 g/kg CP) two weeks post-weaning had a similar effect on diarrhoea as 2500 ppm ZnO compared with a high-protein diet (251 g/kg CP). It can be hypothesized that using medicinal ZnO during the first 14 days post-weaning does not prevent but only postpones the diarrhoea problem. However, two Danish studies have demonstrated reduced diarrhoea frequency in both phase 1 (day 1 to 14) and phase 2 (day 14 to ~24) post-weaning [4,26]. This is consistent with the current trial, where medicinal ZnO had a reducing effect in the PC group in both phase 1, 2 and during the overall trial period. However, the low CP levels in SP and SB did not have a similar reducing effect on diarrhoea treatments as the PC group allocated medicinal ZnO.

On the other hand, in phase 2 the number of diarrhoea treatments were similar between the NC, SP and SB groups, whereas is phase 3 the NC pigs received fewer pen treatments than the SP group (16.1% vs. 29.8%) and numerically fewer than the SB group (21.7%). This is an interesting result, as the pigs received the same CP allocation in phase 3, and neither of the groups received medicinal ZnO. It can therefore be speculated, whether the higher diarrhoea frequency in the SP and SB group are a result of the large increase in CP allocation between phase 2 and 3 (from 176 g/kg to 190 g/kg CP). Whereas the NC group received similar CP allocation in the two phases (187 g/kg to 191 g/kg CP), and their intestine were therefore already adjusted to the high CP level.

Similarly, the current study found no effect of protein source on ADFI or ADG during the overall trial period. This is consistent with a previous study reporting no difference between SBM or SPC post-weaning on ADG [13], whereas Poulsen et al. [14] did report a higher ADG in the SBM group compared with a SPC group (HP 300, Hamlet Protein, Denmark), but no difference between SBM and other SPC groups (Alphasoy, Agilia, Denmark or Vilosoy, Vilomix, Denmark). However, in the study by Poulsen et al. [14] the reduced ADG in the HP 300 SPC group were probably caused by an insufficient supply of Lys. Another study by Yang et al. [27] reported a higher ADG in the SPC group compared to the SBM group from day 0–35 post-weaning. Again, this difference on the effect of protein source on growth performance can be attributed to a difference in CP level between studies, as SPC may have a more positive effect on ADG compared with SBM at higher levels of CP. Yang et al. [27] included 22% CP as-fed in the SPC diet and 21% CP as-fed in the SBM diet from day 0–14 post-weaning, whereas the current study only included 17.6% CP from day 0–14. They further discussed that the CP concentration was highest in the SPC diet, and SPC pigs had a higher ADFI, resulting in more CP and a higher ADG. The present study found no difference in ADFI between SP and SB, but an improved FCR in both SP and SB compared with the NC group. This difference in FCR may be caused by the lower CP levels of the SB and SP groups as well as the difference in AA profile, whereas no difference in FCR between SP and SB was detected. In agreement, Poulsen et al. [14] reported no difference in FCR between SBM and SPC, whereas Yang et al. [27] demonstrated a decreased FCR in SPC pigs from day 0 to 35.

When feeding with extremely low CP levels, the result is an undersupply of the limiting AA and thereby reduced productivity. In the present study, CP levels in the XLA diet were reduced to 15.4% in phase 1 and 15.1% in phase 2, and in order to limit production losses, additional AA (Ile, Leu, His, Phe and Tyr) were added to the diet. Pigs receiving the XLA diet had fewer pen diarrhoea treatments as well as a reduced number of treatment days per pig compared with the NC group. Interestingly, this reduction in diarrhoea treatments was not observed in our previous study [4], where a CP allocation of 14, 17.4, and 19.2% in a three-phase diet did not reduce the number of diarrhoea-treated pens during the nursing period. However, there was a tendency towards fewer treatment days per pig compared to a negative control group without medicinal ZnO (CP levels of 19.1, 18.4, and 18.4%). These results may be caused by an increase in CP levels between phase 1 and phase 2, whereas the current study had a similar CP level in the first two dietary phases. It can be speculated that a low-protein diet decreases the pigs’ susceptibility towards diarrhoea-causing pathogens compared to a high-protein diet. However, a shift in CP level may minimise this positive effect. On the other hand, the current study found no difference in the prevalence of pathogens between diarrhoea outbreaks in the different dietary groups, and in 59% of diarrhoea outbreaks, no pathogens were detected in the faecal pen samples, suggesting that the cause of diarrhoea may not be one of the commonly tested pathogens from this study (*L. intracellularis, B. pilosicoli,* E. coli F4, and E. coli F18), but may be caused by other intestinal factors or pathogens.

Conversely, even with the inclusion of additional AA, the XLA diet decreased ADFI and ADG, resulting in a poorer FCR compared to the four other dietary treatment groups during the nursing period. This may further be due to the different AA profile of the XLA group compared with the PC and NC groups, whereas also the SP and SB groups were allocated the same AA profile as XLA pigs. Consistently, another study on pigs from 10 to 20 kg demonstrated that the CP level can be significantly reduced to 16.8% in a cereal-soybean meal diet without affecting ADG and gain:feed when adding free AA (Lys, Met, Met+Cys, Thr, Trp, Phe, Val, Ile, Leu, and His) [28]. They also demonstrated, that CP levels can be reduced even further to 13.5% without affecting growth performance by adding Val, Ile, Leu, His, and Phe in free form [28]. Feed analysis from the present study revealed a difference between the expected AA inclusion and the analysed contents in all dietary treatments. In particular, the additionally added AA (Leu, Ile, His, Phe and Tyr) was 5 to 12% undersupplied. As pigs from the XLA group had a very low CP level in phase 1 and 2, they were more affected by the low AA allocation compared to pigs from the PC and NC groups, which were allocated standard Danish protein levels [16]. It is therefore likely, that this unintended undersupply in AA may have had a significant effect on the growth performance of the XLA pigs. It is known that AAs regulate several key metabolic pathways that are crucial for the health, maintenance, and growth of the young pig, but also feed intake and absorption of AAs are regulated by the AA availability [29,30]. This suggests that the AA balance was insufficient in the current study, which then negatively affected both the feed intake and growth performance of the XLA pigs. It can also be speculated that the low CP allocation in the XLA diet resulted in an insufficient protein supply to cover the pig’s requirements for non-essential AA [31]. Previous results indicate that when the CP level of feed is below 12%, dietary nitrogen may become the next limiting factor after CP, resulting in insufficient nitrogen available to synthesize non-essential AAs and thereby limiting growth [28]. The unsatisfying results from the XLA group in the current trial may therefore be caused by a combination of a lack of essential AA and a limited amount of nitrogen to cover the synthesis of non-essential AA.

Similar to our previous study by Lynegaard et al. [4], the current study reported that small pigs at weaning (6.0 kg) had fewer diarrhoea treatments than large pigs at weaning (7.8 kg) during the overall trial period from 6 to 30 kg. This demonstrates that small pigs in the current study are not predisposed to diarrhoea post-weaning. It can be speculated that the smaller pigs at weaning were in fact more than 28 days of age at weaning and were held back at the sow unit due to their smaller size. If the small pigs at weaning were older than the larger pigs, they may have had a more mature gastrointestinal tract and were thereby more resistant towards pathogens causing intestinal disorders. At the same time, there is no way of knowing whether the smaller group of pigs had received an AB treatment just prior to entry to the experimental station and were therefore protected against infectious factors causing diarrhoea. On the other hand, smaller pigs at weaning had a reduced ADG and ADFI compared with medium and large pigs throughout the trial period, which is consistent with previous trials from the same experimental station [4,26]. However, small pigs in the current trial had an improved FCR compared with medium and large pigs, indicating that their digestive system was not damaged or altered just by being small at weaning. This is further supported by the low diarrhoea incidence. Moreover, the larger group of pigs at entry had a higher incidence of diarrhoea, which did not seem to have an effect on growth performance. This may be due to the growth-promoting abilities of AB, which would then explain the higher ADG in this group of pigs regardless of diarrhoea. Weaning age was not recorded in the current trial, as the sow unit was too big to facilitate such recordings for the small number of pigs that were sold to the experimental station, which is a limitation of the study.

## 5. Conclusions

No difference in AB diarrhoea treatments and production results was observed when comparing low-protein diets (17.6% CP) based on SBM or on SPC in nursery pigs. Neither of these diets were able to reduce AB diarrhoea treatments at the same level as medicinal zinc. A very low-protein diet (15.4% CP) was able to reduce diarrhoea treatments at least as effectively as the medicinal ZnO in nursery pigs. However, for this diet, an undersupply of amino acids also caused a 42 g/d reduced ADG and 30 g/d reduced ADFI compared to the group of pigs receiving medicinal ZnO in the diet for two weeks post-weaning.

## Figures and Tables

**Table 1:** Analysed chemical composition of the five dietary treatments in the three feeding phases for weaned pigs.

Dietary Treatment ^1^	Phase 1					Phase 2				Phase 3	
	PC	NC	SP	SB	XLA	PC NC	SP	SB	XLA	PC NC	SP SB XLA
Chemical composition											
CP ^2^, g/kg	187.9	188.4	176.0	175.9	155.3	187.2	176.0	176.1	151.4	190.6	190.0
Calcium, g/kg	7.5	8.0	7.3	7.1	7.3	8.0	6.9	7.1	7.8	8.8	8.6
Phosphorous, g/kg	6.5	6.0	6.1	5.0	6.1	6.1	6.1	6.1	6.2	5.4	5.4
Zinc, mg/kg	2740	140	138	166	70	139	138	132	130	144	135
Cobber, mg/kg	150	125	125	122	38	93	96	88	71	74	65
Total amino acids, g/kg											
Lys	12.9	13.6	13.2	13.2	13.0	13.3	13.5	13.1	13.0	13.2	12.8
Met	4.2	4.3	4.2	4.3	4.5	4.0	4.2	4.0	4.4	4.0	3.8
Met + Cys	7.1	7.2	6.9	7.1	6.9	7.0	7.1	6.8	6.8	7.0	6.9
Thr	8.3	8.5	8.3	8.5	8.2	8.4	8.5	8.4	7.9	8.0	8.1
Val	9.4	9.4	8.7	8.9	8.6	9.3	8.7	8.7	8.4	9.0	9.1
His	4.1	4.2	3.8	3.8	3.7	4.3	3.9	4.0	3.6	4.3	4.4
Ile	7.3	7.4	6.7	6.8	6.1	7.3	6.7	6.6	6.2	6.9	7.0
Leu	13.7	13.9	12.6	12.8	12.0	13.6	12.6	12.3	11.9	12.7	12.8
Phe	9.1	9.2	8.4	8.4	7.3	9.1	8.4	8.3	7.3	6.6	8.7
Digestible amino acids ^3^, g/kg											
SID CP	164.3	165.1	154.1	154.0	135.9	164.4	153.9	152.8	155.8	166.7	166.4
SID Lys	11.6	12.3	12.0	12.0	12.0	12.1	12.4	12.2	12.1	12.0	11.6
SID Met	3.9	3.9	3.9	4.0	4.3	3.7	4.0	3.7	4.1	3.7	3.6
SID Met + Cys	6.2	6.3	6.1	6.3	6.2	6.2	6.2	6.0	6.1	6.2	6.1
SID Thr	7.3	7.4	7.4	7.5	7.4	7.4	7.5	7.3	7.1	7.0	7.1
SID Val	8.1	8.1	7.5	7.6	7.5	8.1	7.5	7.5	7.4	7.8	7.9
SID His	3.6	3.7	3.3	3.3	3.2	3.7	3.4	3.5	3.2	3.7	3.8
SID Ile	6.4	6.5	5.8	5.8	5.4	6.3	5.9	5.7	5.5	6.1	6.1
SID Leu	12.2	12.3	11.2	11.3	10.7	11.9	11.0	10.8	10.5	11.2	11.3
SID Phe	8.1	8.1	7.5	7.5	6.4	8.2	7.4	7.3	6.4	7.7	7.8

^1^ PC = positive control; NC = negative control, SP = soy protein concentrate, SB = soybean meal, XLA = X-low-protein + amino acids; ^2^ CP = crude protein; ^3^ SID = standardized ileal digestible: the contents of SID CP and amino acids were calculated based on analysed total values of the six dietary treatments and on SID digestibility coefficients of the feed ingredients from Danish Agro (Sjölund, Denmark).

**Table 2:** Actual dietary concentrations of crude protein (CP) and amino acids relative to expected amounts in the five dietary treatments during the three feeding phases for weaned pigs.

Dietary Treatment ^1^	Analysed Content, % of Expected Level										
	Phase 1					Phase 2				Phase 3	
	PC	NC	SP	SB	XLA	PC NC	SP	SB	XLA	PC NC	SP, SB XLA
Standardized ileal digestible ^2^											
CP	98	98	100	100	101	100	101	99	101	100	100
Lys	94	100	98	98	98	100	103	102	101	102	95
Met	91	95	93	95	96	95	98	93	95	97	92
Met + Cys	94	95	92	95	93	94	94	91	91	93	91
Thr	97	99	99	100	99	100	101	99	96	97	95
Val	103	103	103	103	100	103	101	103	100	104	103
His	92	95	94	94	91	95	100	100	94	90	95
Ile	91	93	95	95	93	95	102	98	96	95	97
Leu	95	96	98	99	97	98	98	101	97	95	97
Phe	96	96	101	101	97	101	103	100	98	96	99

^1^ PC = Positive control; NC = Negative control, SP = Soy protein concentrate, SB = Soybean meal, XLA = X-low-protein + amino acids. ^2^ Standardized ileal digestible = SID: The content of SID CP and amino acids were calculated based on analysed total values of the six dietary treatments and on SID digestibility coefficients of the feed ingredients from Danish Agro (Sjölund, Denmark).

**Table 3:** Diarrhoea-related antibiotics treatments in the five dietary groups during the nursing period in pigs.

Item	Dietary Treatment ^1^					SEM	*p*-Value
	PC	NC	SP	SB	XLA		
Pens, n	75	148	75	75	75		
Pigs, n	948	1866	949	948	927		
Diarrhoea pen treatments, %							
Phase 1, 6–9 kg	0.0 (0; 100)	6.7 (0.2; 68.5)	4.0 (0.009; 95.0)	4.0 (1.4; 10.6)	0.0 (0; 100)	-	NS
Phase 2, 9–15 kg	26.7 ^b^ (17.9; 37.8)	44.6 ^a^ (36.8; 52.7)	33.3 ^a^ (23.6; 44.70)	28.0 ^a^ (19.0; 39.2)	4.0 ^b^ (1.3; 11.7)	-	<0.001
Phase 3, 15–30 kg	9.8 ^a^ (4.9; 18.7)	16.1 ^a^ (11.0; 23.0)	29.8 ^b^ (20.4; 41.3)	21.7 ^a^ (13.7; 32.6)	16.2 ^a^ (9.5; 26.4)	-	0.027
Total period, 6–30 kg	31.8 ^b^ (22.2;43.1)	53.5 ^a^ (45.4; 61.4)	49.3 ^a^ (38.2; 60.1)	41.3 ^a^ (29.5; 51.4)	21.1 ^b^ (13.3; 31.8)	-	<0.001
Treatment days per pig							
Phase 1, 6–9 kg	0.04 ^b^	0.39 ^a^	0.26 ^a^	0.32 ^a^	0.05 ^b^	0.10	0.017
Phase 2, 9–15 kg	1.82 ^b^	3.05 ^a^	2.15 ^b^	1.98 ^b^	0.38 ^b^	0.32	<0.001
Phase 3, 15–30 kg	0.73 ^a^	1.15 ^a^	1.94 ^b^	1.42 ^a^	1.27 ^a^	0.28	0.042
Total period, 6–30 kg	2.6 ^b^	4.6 ^a^	4.3 ^a^	3.7 ^a^	1.7 ^b^	0.47	<0.001

^1^ PC = positive control; NC = negative control; SP = soy protein concentrate; SB = soybean meal; XLA = X-low-protein + amino acids. ^a,b^ Values within a row with different superscripts differ significantly at *p* < 0.05 in relation to group NC; NS = not significant, n = number.

**Table 4:** Production results in weaned pigs receiving different diets.

Item	Dietary Treatment ^1^					SEM	*p*-Value
	PC	NC	SP	SB	XLA		
Pigs, n	948	1 866	949	948	927		
BW at entry	6.9	6.9	6.9	6.9	6.9	0.09	NS
BW at exit	30.7 ^a^	30.7 ^a^	30.8 ^a^	30.7 ^a^	29.2 ^b^	0.23	<0.001
Phase 1, 6–9 kg							
Days per pig	11	11	11	11	11		
ADFI ^1^, g/day	168 ^a^	173 ^ab^	178 ^a b^	181 ^b^	175 ^ab^	0.004	0.009
ADG ^1^, g/day	132 ^a^	128 ^a^	127 ^a^	131 ^a^	108 ^b^	4.00	<0.001
FCR1, kg/kg growth	1.33 ^a^	1.42 ^ab^	1.46 ^b^	1.44 ^a b^	1.67 ^c^	0.035	<0.001
Phase 1+2, 6–15 kg							
Days per pig	30	30	30	30	30		
ADFI, g/day	396 ^ab^	387 ^b^	393 ^ab^	404 ^a^	359 ^c^	0.005	<0.001
ADG, g/day	294 ^a^	286 ^a^	280 ^a^	290 ^a^	217 ^b^	4.36	<0.001
FCR, kg/kg growth	1.36 ^a^	1.36 ^a^	1.41 ^b^	1.40 ^b^	1.66 ^c^	0.008	<0.001
Overall period, 6–30 kg							
Days per pig	48	48	48	48	49		
ADFI, g/day	680 ^a^	680 ^a^	690 ^a^	700 ^a^	650 ^b^	0.006	<0.001
ADG, g/day	480 ^a^	477 ^a^	480 ^a^	482 ^a^	438 ^b^	4.76	<0.001
FCR, kg/kg growth	1.42 ^a^	1.43 ^ab^	1.44 ^b c^	1.45 ^c^	1.49 ^d^	0.007	<0.001
Extracted, %	4.5 ^ab^ (3.3;6.2)	5.5 ^a^ (4.4;6.8)	3.2 ^b^ (2.2;4.6)	3.5 ^b^ (2.5;5.0)	3.5 ^b^ (2.4;5.0)	-	0.023
Dead, %	1.0 (0.5;1.9)	1.0 (0.6;1.6)	0.6 (0.3;1.4)	1.0 (0.6;2.0)	0.5 (0.2;1.2)	-	0.516

^1^ PC = Positive control; NC = Negative control, SP = Soy protein concentrate, SB = Soybean meal, XLA = X-low-protein + amino acids; ADG = average daily gain, ADFI = average daily feed intake, FCR = feed conversion ratio. ^a,b,c^ Values within a row with different superscripts differ significantly at *p* < 0.05; NS = not significant.

**Table 5:** The effect of pig entry bodyweight (small, medium, large) on diarrhoea treatments and growth performance in the overall trial period (6–30 kg).

Item	Bodyweight Group at Entry			SEM	*p*-Value
	Small	Medium	Large		
Pens, n	144	144	160		
BW at entry	6.0	6.8	7.8		
Diarrhoea treated pens, % ^1^	32.7 ^a^ (25.4;41.2)	36.3 ^ab^ (28.6;44.8)	45.4 ^b^ (37.5;53.6)	-	0.074
ADG ^2^, g/day	451 ^a^	463 ^b^	491 ^c^	5.78	<0.001
ADFI ^2^, kg/day	0.65 ^a^	0.67 ^b^	0.71 ^c^	0.007	<0.001
FCR ^2^, kg/kg growth	1.43 ^a^	1.45 ^b^	1.45 ^b^	0.009	0.008

^1^ Accumulated treatments = Each pen is only counted once. ^2^ ADG = average daily gain, ADFI = average daily feed intake, FCR = feed conversion ratio. ^a,b,c^ Values within a row with different superscripts differ significantly at *p* < 0.05.

## Data Availability

Data are available upon request.

## Supplementary Materials

The following are available online at https://www.mdpi.com/2076-2615/11/3/678/s1, Table S1: Composition of the five dietary treatments for weaned pigs in phase 1, Table S2: Composition of the five dietary treatments for weaned pigs in phase 2, Table S3: Composition of the five dietary treatments for weaned pigs in phase 3, Table S4: Expected nutritional contents of the five dietary treatments in the three feeding phases for weaned pigs.
