# Supplementary material for: A Very Low CP Level Reduced Diarrhoea and Productivity in Weaner Pigs, but No Differences between Post-Weaning Diets Including Soybean Meal or Soy Protein Concentrate Were Found
Source: Animals (Basel). 2021 Mar 4;11(3):678. doi: 10.3390/ani11030678 (PMC7998764; doi:10.3390/ani11030678)
Supplement: Supplementary file 1 [file animals-11-00678-s001.pdf]

A very low CP level reduced diarrhoea and productivity in weaned pigs, but no differences between post-weaning diets including soybean meal or soy protein concentrate were found[1]

Julie C. Lynegaard, Niels J. Kjeldsen, Julie K. Bache, Nicolai R. Weber, Christian F. Hansen, Jens Peter Nielsen and Charlotte Amdi

Supplementary materials

Table S1. Composition of the five dietary treatments for weaned pigs in phase 1.

	Dietary treatment[1]				
Item (%)	PC	NC	SP	SB	XLA
Wheat	46.6	47.7	53.0	51.7	59.4
Barley	20.0	20.0	20.0	20.0	20.0
Soybean meal	7.0	7.0	0.5	7.0	0.5
Soy protein concentrate[2]	6.5	6.4	7.5	2.2	0
Potato protein conc.	4.0	4.0	4.0	4.0	4.0
Fishmeal	2.0	2.0	2.0	2.0	2.0
Whey powder	6.0	6.0	6.0	6.0	6.0
Fatty acid distillate	2.4	2.1	1.7	1.9	1.2
Monocalcium phosphate	1.4	1.2	1.3	1.3	1.5
Sodium chloride	0.6	0.6	0.6	0.6	0.6
Sodium bicarbonate	0.1	0.1	0.1	0.1	0.1
Lysine sulphate 70%	0.69	0.69	0.90	0.86	1.25
Methionine 98%	0.11	0.11	0.15	0.15	0.23
Threonine 98%	0.13	0.13	0.20	0.20	0.33
Tryptophan 99%	0.05	0.05	0.07	0.07	0.12
Valin 96,5%	0.03	0.03	0.05	0.05	0.20
Mineral-vitamin premix[3]	0.40	0.40	0.40	0.40	0.40
Phytase[4]	0.03	0.03	0.03	0.03	0.03
Benzoic acid	0.50	0.50	0.50	0.50	0.50
Calcium formate	1.00	1.00	1.00	1.00	1.00
Microgrits Green[5]	0.05	0	0	0	0
Microgrits Blue[5]	0	0	0.05	0	0
Zinc oxide	0.30	0	0	0	0
Isoleucine 98,5%	0	0	0	0	0.12
Leucine 98,5%	0	0	0	0	0.22
Histidine 98,5%	0	0	0	0	0.08
Phenylalanine 98,5%	0	0	0	0	0.08
Tyrosine 98,5%	0	0	0	0	0.15

[1]PC = Positive control with medicinal zinc oxide; NC = Negative control without medicinal zinc oxide, SP = Soy protein concentrate, SB = Soybean meal, XLA = X-low protein + amino acids.
[2]Vilosoy from Vilomix (Sjølund, Denmark).
[3]DA Vit weaning mix from Danish Agro (Sjølund, Denmark), DA = Danish Agro.
[4]DSM Ronozyme, phytase enzyme from DSM (Brøndby, Denmark).
[5]Microgrits were added to the diets, to ensure that the right feed was delivered from the right silos.

Table S2. Composition of the five dietary treatments for weaned pigs in phase 2.

	Dietary treatment[1]			
Item	PC + NC	SP	SP	XLA
Wheat	52.1	59.1	54.9	65.4
Barley	20.0	20.0	20.0	20.0
Soybean meal	14.0	6.0	14.0	2.2
Soy protein concentrate[2]	2.9	2.6	0.85	0
Potato protein conc.	3.0	3.0	2.0	3.0
Fishmeal	0	2.0	0	0.5
Fatty acid distillate	2.7	1.9	2.5	1.5
Monocalcium phosphate	0.2	0.1	0.2	0.1
Sodium chloride	1.3	1.2	1.3	1.5
Sodium bicarbonate	0.6	0.6	0.6	0.6
Lysine sulphate 70%	0.1	0.1	0.1	0.1
Methionine 98%	0.76	0.93	0.93	1.35
Threonine 98%	0.13	0.15	0.17	0.24
Tryptophan 99%	0.16	0.22	0.23	0.36
Valin 96,5%	0.05	0.07	0.06	0.12
Mineral-vitamin premix[3]	0.05	0.07	0.08	0.23
Phytase[4]	0.40	0.40	0.40	0.40
Benzoic acid	0.03	0.03	0.03	0.03
Calcium formate	0.50	0.50	0.50	0.50
Microgrits Green[5]	1.00	1.00	1.00	1.00
Microgrits Blue[5]	0.05	0	0	0
Zinc oxide	0	0.05	0	0
Isoleucine 98,5%	0	0	0	0.14
Leucine 98,5%	0	0	0	0.26
Histidine 98,5%	0	0	0	0.09
Phenylalanine 98,5%	0	0	0	0.1
Tyrosine 98,5%	0	0	0	0.17

[1]PC = Positive control with medicinal zinc oxide; NC = Negative control without medicinal zinc oxide, SP = Soy protein concentrate, SB = Soybean meal, XLA = X-low protein + amino acids.
[2]Vilosoy from Vilomix (Sjølund, Denmark).
[3]DA Vit weaning mix from Danish Agro (Sjølund, Denmark), DA = Danish Agro.
[4]DSM Ronozyme, phytase enzyme from DSM (Brøndby, Denmark).
[5]Microgrits were added to the diets, to ensure that the right feed was delivered from the right silos.

Table S3. Composition of the five dietary treatments for weaned pigs in phase 3.

	Dietary treatment[1]	
Item	PC + NC	SP + SB + XLA
Wheat	49.8	49.7
Barley	20.0	20.0
Soybean meal	21.0	22.5
Soy protein concentrate[2]	2.1	0.5
Fatty acid distillate	1.9	2.0
Limestone	1.5	1.5
Monocalcium phosphate	0.9	0.9
Sodium chloride	0.5	0.5
Sodium bicarbonate	0.1	0.1
Lysin sulphate 70%	0.72	0.81
Methionine 98%	0.14	0.16
Threonine 98%	0.17	0.21
Tryptophan 99%	0.03	0.04
Valin 96,5%	0.06	0.09
Mineral-vitamin premix[3]	0.40	0.40
Phytase[4]	0.03	0.03
Benzoic acid	0.50	0.50
Microgrits Green[5]	0.05	0
Microgrits Blue[5]	0	0.05

[1]PC = Positive control with medicinal zinc oxide; NC = Negative control without medicinal zinc oxide, SP = Soy protein concentrate, SB = Soybean meal, XLA = X-low protein + amino acids.
[2]Vilosoy from Vilomix (Sjølund, Denmark).
[3]DA Vit weaning mix from Danish Agro (Sjølund, Denmark), DA = Danish Agro.
[4]DSM Ronozyme, phytase enzyme from DSM (Brøndby, Denmark).
[5]Microgrits were added to the diets, to ensure that the right feed was delivered from the right silos.

Table S4. Expected nutritional contents of the five dietary treatments in the three feeding phases for weaned pigs.

	Phase 1					Phase 2				Phase 3	
Dietary treatment[1]	PC	NC	SP	SB	XLA	PC NC	SP	SB	XLA	PC NC	SP SB XLA
Chemical composition											
Energy, FEsv/kg[2]	1.16	1.16	1.16	1.16	1.16	1.14	1.14	1.14	1.14	1.11	1.11
ME MJ/kg	14.2	14.2	14.2	14.2	14.2	13.9	13.9	13.9	13.9	13.5	13.5
CP, g/kg	191.6	191.9	176.3	176.5	153.5	188.9	174.3	175.9	150.5	190.9	190.6
Crude fibre, g/kg	27.6	27.6	27.1	27.4	26.5	30.6	29.3	30.3	28.6	32.2	32.3
Calcium, g/kg	7.5	7.1	7.1	7.1	7.1	6.9	6.9	7.8	6.9	8.4	8.4
Phosphorous, g/kg	6.4	5.9	5.9	5.1	5.9	5.8	5.8	5.9	5.9	5.2	5.2
Zinc, mg/kg	2500	0	0	0	0	0	0	0	0	0	0
Cobber, mg/kg	0	0	0	0	0	0	0	0	0	0	0
Total amino acids, g/kg											
Lys	13.7	13.7	13.5	13.4	13.2	13.4	13.2	13.4	12.9	13.0	13.4
Met	4.3	4.3	4.5	4.5	4.8	4.2	4.4	4.3	4.7	4.1	4.2
Met + Cys	7.6	7.6	7.6	7.5	7.5	7.5	7.5	7.5	7.5	7.5	7.5
Thr	8.7	8.7	8.6	8.6	8.5	8.5	8.4	8.6	8.3	8.3	8.6
Val	9.2	9.2	8.5	8.6	8.6	9.1	8.6	8.5	8.5	8.8	8.9
His	4.5	4.5	4.0	4.0	4.0	4.5	4.0	4.1	3.9	4.6	4.6
Iso	7.9	7.9	7.0	7.0	6.6	7.5	6.7	6.7	6.5	7.3	7.2
Leu	14.4	14.4	12.9	13.0	12.5	13.9	12.5	12.5	12.3	13.4	13.3
Phe	9.3	9.3	8.4	8.4	7.5	9.2	8.1	8.3	7.4	9.0	8.9
Digestible amino acids[3], g/kg											
SID CP	167.9	168.1	154.2	154.3	134.3	165.1	152.2	153.6	131.4	167.1	166.8
SID Lys	12.3	12.3	12.2	12.2	12.2	12.1	12.0	12.0	12.0	11.8	12.2
SID Met	4.1	4.1	4.2	4.2	4.5	3.9	4.1	4.0	4.3	3.8	3.9
SID Met + Cys	6.6	6.6	6.6	6.6	6.7	6.6	6.6	6.6	6.7	6.7	6.7
SID Thr	7.5	7.5	7.5	7.5	7.5	7.4	7.4	7.4	7.4	7.2	7.5
SID Val	7.9	7.9	7.3	7.4	7.5	7.9	7.4	7.3	7.4	7.5	7.7
SID His	3.9	3.9	3.5	3.5	3.5	3.9	3.4	3.5	3.4	4.1	4.0
SID Iso	7.0	7.0	6.1	6.1	5.8	6.6	5.8	5.8	5.7	6.4	6.3
SID Leu	12.8	12.8	11.4	11.4	11.0	12.2	11.2	10.7	10.8	11.8	11.7
SID Phe	8.4	8.4	7.4	7.4	6.6	8.1	7.2	7.3	6.5	8.0	7.9

[1]PC = Positive control with medicinal zinc oxide; NC = Negative control without medicinal zinc oxide, SP = Soy protein concentrateSoybean meal, XLA = X-low protein + amino acids.

[2]FEsv = Danish Feed Units, which are potentially physiological energy closely related to net energy.
[3] Standardized ileal digestible = SID: The content of SID CP and amino acids were calculated based on analysed total values of the six dietary treatments and on SID digestibility coefficients of the feed ingredients from Danish Agro (Sjölund, Denmark).
